# Mortality Dynamics of *Spodoptera frugiperda* (Lepidoptera: Noctuidae) Immatures in Maize

**DOI:** 10.1371/journal.pone.0130437

**Published:** 2015-06-22

**Authors:** Andrea Corrêa Varella, Alexandre Carlos Menezes-Netto, Juliana Duarte de Souza Alonso, Daniel Ferreira Caixeta, Robert K. D. Peterson, Odair Aparecido Fernandes

**Affiliations:** 1 Montana State University, Department of Plant Sciences and Plant Pathology, Bozeman, Montana 59717, United States of America; 2 UNESP—Univ Estadual Paulista, Departamento de Fitossanidade, Jaboticabal, SP, 14884–900, Brazil; 3 Embrapa Arroz e Feijão, Santo Antônio de Goiás, GO, 75375–000, Brazil; 4 Montana State University, Department of Land Resources & Environmental Sciences, Bozeman, Montana 59717, United States of America; Pennsylvania State University, UNITED STATES

## Abstract

We characterized the dynamics of mortality factors affecting immature developmental stages of the fall armyworm, *Spodoptera frugiperda* (J.E. Smith) (Lepidoptera: Noctuidae). Multiple decrement life tables for egg and early larval stages of *S*. *frugiperda* in maize (*Zea mays *L.) fields were developed with and without augmentative releases of *Telenomus remus* Nixon (Hymenoptera: Platygastridae) from 2009 to 2011. Total egg mortality ranged from 73 to 81% and the greatest egg mortality was due to inviability, dislodgement, and predation. Parasitoids did not cause significant mortality in egg or early larval stages and the releases of *T*. *remus *did not increase egg mortality. Greater than 95% of early larvae died from predation, drowning, and dislodgment by rainfall. Total mortality due to these factors was largely irreplaceable. Results indicate that a greater effect in reducing generational survival may be achieved by adding mortality to the early larval stage of *S*. *frugiperda*.

## Introduction

The fall armyworm (FAW), *Spodoptera frugiperda* (J.E. Smith) (Lepidoptera: Noctuidae), is a polyphagous noctuid and one of the more economically important pests in the Americas [1, 2]. Outbreaks occur regularly in maize (*Zea mays* L.), resulting in populations that often reach the economic injury level [3]. In Brazil, although the main maize growing season occurs in the summer (wet season), in the south-central region, growers have taken advantage of the late-season summer rainfalls to cultivate a second maize crop which lasts until early winter (dry season). This new growing season regime, in addition to FAW’s high reproductive rate, multivoltinism, broad host range [1], and ability to migrate long distances [4], significantly increases FAW’s outbreak potential and subsequent yield losses in maize [5].

Chemical management techniques adopted to date have not provided adequate control and insecticide resistance has been reported [6]. Therefore, biological control techniques are being developed to manage FAW. Egg parasitoids such as *Telenomus remus* Nixon (Hymenoptera: Platygastridae) and *Trichogramma* spp. (Hymenoptera: Trichogrammatidae) have been used as biological control agents in Venezuela [7] and Colombia [8]. In Brazil, studies regarding identification and quantification of FAW’s natural mortality sources in maize fields have been conducted [9–11], but these studies do not provide an overall understanding of the mortality dynamics of FAW populations (i.e., how the levels and patterns of cohort mortality change or may change temporally and spatially) [12].

An ecological approach using demographic analytical methods can provide insights into the basic population ecology of FAW and can allow us to identify mortality agents that might have the potential to be used effectively in biological control programs. Moreover, life-table approaches permit a better understanding of the timing and magnitude of mortality factors, which allows for the assessment of the role each factor plays in population suppression [13]. The background information provided by life-table studies can be used for developing sustainable management strategies for managing FAW. Because of the potential for the commercial use of *T*. *remus* in Brazil [14], it is also important to assess the mortality dynamics of FAW in the presence and absence of this biological control agent. Therefore, the aim of this study was to construct partial cohort-based life tables for FAW in maize fields, with and without mass release of *T*. *remus*, to understand the dynamics of mortality factors affecting egg and early larval stages.

## Materials and Methods

### Field plots

Studies were conducted in maize during the wet (first) and dry (second) seasons in 2009, 2010, and 2011 in two fields (0.70 ha) located at the São Paulo State University Research Farm (Jaboticabal/SP, Brazil) (21°14’S and 48°17’W). The University Research Farm is located approximately 4 km from the nearest city and is surrounded mostly by sugarcane (*Saccharum* spp.), and small (5–25 ha) cotton (*Gossypium* sp.) and maize fields. According to the Köppen Classification System, climatic conditions of Jaboticabal municipality are grouped in Aw climate, which is characterized by wet summer and dry winter seasons [15]. Meteorological readings on temperature, relative humidity, and precipitation for all six growing seasons were obtained from São Paulo State University Weather Station, which was located about 350 meters from the experimental fields (Table 1).

**Table 1:** Weather conditions during six maize growing seasons in Jaboticabal, São Paulo, Brazil.

Year/Season	Season	Temperature (C°)		Relative Humidity (%)		Daily Precipitation (mm)	
	characteristic	Mean	Range	Mean	Range	Mean	Range
2009/1^st^	Wet	24.13	14.7–34.9	79.43	50.4–94.2	7.82	0.0–70.4
2009/2^nd^	Dry	22.43	14.8–35.5	77.07	30.1–94.8	3.41	0.0–77.4
2010/1^st^	Wet	24.60	18.0–34.8	80.20	66.2–97.0	8.51	0.0–53.2
2010/2^nd^	Dry	22.10	11.7–33.7	74.90	62.8–96.3	3.14	0.0–58.3
2011/1^st^	Wet	24.51	17.0–33.4	78.30	63.1–93.3	7.69	0.0–75.4
2011/2^nd^	Wet	21.83	10.1–33.7	78.53	57.2–96.1	6.43	0.0–80.7

Conventional hybrid maize (Dow 2B688, Dow AgroSciences) was planted on 10 December 2008, 23 March 2009, 17 December 2009, 1 March 2010, 25 November 2010, and 24 February 2011, in two fields (ca. 0.3 ha each). Crops were managed according to standard agronomic practices [16] and no insecticides were applied. In each field, two plots measuring 50 x 15 m each were chosen randomly located on separate ends of the same maize field to allow the release of *T*. *remus*. Thus, the treatments consisted of egg parasitoid release in one plot at one end of the field and non-release in one plot at the other end of the field. The experiment was replicated twice every season. The distance between plots was greater than 100 m to avoid parasitoid dispersal to non-release sites, because this parasitoid species disperses no further than 19 m (data not published). Experiments with larvae were performed only in one maize field per season. Experiments were conducted when maize was in early- to mid-whorl stage.

### Insects

Colonies of FAW and *T*. *remus* were established at the end of 2008 with non-parasitized and parasitized FAW egg masses received from the Maize and Sorghum Research Center (Embrapa Maize and Sorghum—Sete Lagoas, Minas Gerais, Brazil) where the *T*. *remus* colony has been in laboratory conditions for more than 20 years. Fall armyworm eggs and larvae used in the experiments were taken from these laboratory colonies. The colony of *T*. *remus* was maintained on FAW eggs. Rearing conditions were 25±1°C, 70±10% RH, and 12:12 h (L:D) [17].

### Cohort establishment

The age of insects in field populations is usually difficult to determine [13], and the overlap of FAW generations makes identification of single cohorts even more difficult. Therefore, cohorts of eggs and larvae were established separately. In each plot, cohorts of eggs were established using 20 sentinel egg masses (< 24-h-old) of approximately 230 eggs each. Paper sheets containing egg masses were collected from the FAW colony and cut to individualize egg masses. Then, egg masses were stapled on the newly emerged whorl leaf of maize plants (one egg mass/plant) at random locations in the field. To quantify the initial number of eggs, egg masses were photographed and the eggs were counted by using the images. The use of this computational method was necessary because egg masses usually contain more than one layer of eggs and each layer needed to be counted separately and then added to obtain the total number of eggs. Initially we counted the eggs on the uppermost layer. As eggs were counted, they were individually marked with a dot using the software Paintbrush for Windows (Microsoft Corporation, Richmond, Washington, USA). Eggs of the underneath layers are lined with those on the uppermost. Thus, for the eggs below the uppermost layer, we counted the visible fringe eggs and added to the number of eggs already counted on the immediate upper layer. Therefore, the number of eggs on an underneath layer was determined by summing the number of visible eggs in this layer to the number of eggs from the immediately superior layer, as suggested by Leuck and Perkins [18].

To obtain larval cohorts, paper sheets containing egg masses were removed from the colony and glued individually into small plastic cups (100 ml) with lids. Two to four hours after larval eclosion, the plastic cups were taken to the field, opened, and stapled on the newly emerged whorl leaf of 70 maize plants (one plastic cup containing an egg mass/plant). To prevent larval dispersal, plants within 1.5 m from the infested plant were removed. All naturally occurring eggs and larvae of FAW were manually removed from plants before release of the larvae. To assess the initial number of eggs, egg masses were photographed and the eggs were counted similarly as above. Ten percent of the egg masses were kept in the laboratory to assess egg viability. The initial number of larvae was estimated by subtracting the average number of inviable eggs from the initial number of eggs.

### Releases of *T*. *remus*

Fall armyworm egg masses were removed from the colony, glued on cardboard squares (7 cm x 6 cm), and offered to the *T*. *remus* colony for 24 h. Then, the cardboard squares were transferred to plastic cups and kept under controlled conditions (25±1°C, 70±10% RH, and L12:D12) until parasitoid emergence. On each release site, two cardboard squares were used. Approximately 15,000 insects were released per plot during early morning of the first experimental day [19]. This parasitoid density is equivalent to 200,000 parasitoids/ha, which is similar to densities used in *T*. *remus* release in maize fields in Colombia [8] and Venezuela [7]. The sex ratio of *T*. *remus* raised on FAW eggs at 25°C is ca. 0.6 (60% female: 40% male), as observed by Bueno et al. [20]. The release procedure consisted of walking through each row of maize with plastic cups containing adults opened and near the plant’s whorl [21]. By the end of this procedure, cups containing parasitoids were fixed in two release points in the center of the plot for 24 hours to allow the release of the remaining individuals. All parasitoids were 24-h old and had been previously fed *ad libitum* with honey [22].

### Determination of mortality factors

Two experiments with eggs were performed per season, except for the first season of 2009 when no experiments with eggs took place. Natural mortality factors were observed in eight 2-h-long diurnal and nocturnal observations during three days after infestation (= embryonic period). Insects interacting with the egg masses were photographed and collected for identification. On the third day, egg masses were retrieved and placed into Petri dishes containing artificial diet [23]. They were held at room temperature in the laboratory until eclosion of FAW or emergence of adult parasitoids. Egg mortality factors were predation, parasitism, dislodgment, inviability, desiccation, and unknown. Predation was characterized by the presence of chorion remaining on the paper sheet containing an egg mass. Dislodgement was due to rain and wind. Dislodged eggs were counted as dead, but it is possible that some of them survived and successfully produced larvae. Inviable eggs appeared normal, but failed to hatch. Desiccated eggs were intact, but dried and shriveled and fail to hatch. When cause of death could not be determined, it was designated as ‘unknown’.

One larval experiment per season was performed. Each day, 10 infested plants were randomly chosen and covered individually with plastic trash bags, cut at the soil level, and taken to the laboratory for observation and collection of larvae. Collected larvae were placed in Petri dishes containing artificial diet. They were held at room temperature in the laboratory until pupation or emergence of parasitoids. Larval mortality factors comprised two mortality categories: parasitism and other factors.

### Life table analysis

The construction of multiple decrement life tables for eggs and early larvae was performed using calculation techniques described by Carey [24] and Peterson et al. [25]. Variables are defined as: x—the life stage index, l_x_—the number of individuals entering the stage, d_x_—the number of individuals dying in a stage, al_x_—the fraction of the cohort living at the beginning of the stage, ad_ix_—fraction of the original cohort dying in stage x due to cause i, ad_x_—fractions of deaths in stage x from all causes, and aq_x_—the probability of dying in stage x in the presence of all causes.

Additionally, the irreplaceable mortality was estimated for each mortality factor and stage using an elimination-of-cause method that examines combinations of causes of death in relationship to the absence of other causes [25, 26]. As the term suggests, irreplaceable mortality is the mortality rate from a specific factor that cannot be replaced by another factor in the system being studied [27]. This particular concept, and the multiple decrement life table more generally, emerged from reliability and competing risk theory in operations research and has the putative broad assumption that an individual’s surviving to a certain age is the mathematical product of all independent risk probabilities [24]. More specific assumptions include: (i) each death is due to a single cause; (ii) each individual in a population has the same probability of dying from any of the causes operating in the population; and (iii) the probability of dying from any given cause is independent of the probability of dying from any other source [24].

The elimination-of-cause method was used as part of the spreadsheet program M-DEC [28] and allowed to visualize factors that could compensate for the mortality of FAW in the absence of another factor. Carey [24] derived a quadratic solution to attribute mortality to each individual factor in the absence of all other factors, and we use his quadratic solution for this analysis within M-DEC [28]. Elimination-of-cause analysis relies on the probability of surviving each source of mortality (*P*_*x*_) and its complement (1 − *q*_*x*_) where (1 − *q*_*1*_) *x*…*x* (1 − *q*_*n*_) is the chance of jointly surviving a set of mortality factors and its complement, 1 − [(1 − *q*_*1*_) *x*…*x* (1 − *q*_*n*_)], is the chance of jointly dying from a set of mortality factors. To estimate mortality in the absence of one or more factors, two simultaneous equations with two unknowns are used. For example, by expressing *q*_*1*_ in terms of *q*_*2*_, *D*_*1*_, and *D*_*2*_ (the fraction of all individuals observed that died of cause 1 and 2), this yields the quadratic equation *aq*_*2*_^*2*^ + *bq*_*2*_ + *c* = 0, where *a* = *D*_*1*_, *b* = − (*D*_*1*_ +*D*_*2*_) and *c* = *D*_*2*_(*D*_*1*_ + *D*_*2*_). The value of *q*_*2*_ can be found by substituting *a*, *b*, and *c* into the quadratic formula.

### Data analysis

Analysis of variance was conducted to compare the percentage of predation and parasitism in fields with and without *T*. *remus* release using SAS PROC MIXED [29].

## Results

### Egg mortality

Partial multiple decrement life tables were developed for cohorts of eggs from non-release and release sites (Table 2). Egg masses evaluated in this study contained, on average, 237 eggs. The fate of several thousand individual eggs was evaluated in each growing season (S1 Table). Results show that total mortality did not vary considerably among years. The percentage of deaths from all causes ranged from 73 to 81%. Inviability was the main mortality factor in the 2009 and 2010 seasons (Table 2). In 2011, predation and dislodgment were the main mortality factors during the first and second seasons, respectively (Table 2).

**Table 2:** Multiple decrement life table for egg stage of FAW in maize fields with and without *Telenomus remus* releases.

Year/ Season	aq_x_	al_x_	Predation	Parasitism	Dislodgement	Inviability	Desiccation	Unknown
**Without *T*. *remus* release**								
2009/2^nd^	0.741	1	0.181	0.012	0.051	0.472	0.022	0.003
2010/1^st^	0.742	1	0.068	0.011	0.272	0.327	0.044	0.020
2010/2^nd^	0.814	1	0.256	0.021	0.068	0.439	0.008	0.022
2011/1^st^	0.761	1	0.365	0.033	0.105	0.251	0.002	0.005
2011/2^nd^	0.736	1	0.175	0.001	0.449	0.095	0.002	0.014
**With *T*. *remus* release**								
2009/2^nd^	0.813	1	0.139	0.090	0.056	0.497	0.028	0.002
2010/1^st^	0.735	1	0.069	0.014	0.267	0.283	0.070	0.030
2010/2^nd^	0.776	1	0.253	0.064	0.040	0.372	0.012	0.035
2011/1^st^	0.733	1	0.336	0.029	0.066	0.271	0.003	0.027
2011/2^nd^	0.755	1	0.122	0.048	0.477	0.083	0.011	0.015

aq_x_—the probability of dying in stage x in the presence of all causes; al_x_—the fraction of the cohort living at the beginning of the stage; aq_ix_—the probability of dying in stage x in the presence of cause i (1: predation, 2: parasitism, 3: dislodgement, 4: inviability, 5: desiccation, and 6: unknown).

The probability of dying from predation and dislodgment varied considerably among seasons. In dry weather conditions, egg mortality due to predation ranged from 14 to 25% and dislodgment was responsible for 5 to 7% of egg mortality. In wet weather conditions, 26 to 47% of eggs were dislodged and predators consumed 6 to 36% of eggs. The percentage of eggs lost due to predation was not significantly different (F_1,30_ = 0.12; *P* = 0.73) among plots with (44.29% ± 5.21) and without (45.98% ± 4.23) parasitoid releases. *Doru luteipes* Scudder (Dermaptera: Forficulidae), *Harmonia axyridis* (Pallas), and *Eriopis connexa* (Germar) (Coleoptera: Coccinelidae) were observed feeding on egg masses. Also, common herbivores associated with maize such as *Diabrotica speciosa* (Germar) (Coleoptera: Chrysomelidae), *Monocrepidius* aff. *posticus* (Eschscholtz) and *Monocrepidius fuscofasciatus* (Eschscholtz) (Coleoptera: Elateridae), and *Leptoglossus zonatus* (Dallas) (Hemiptera: Coreidae) were associated with egg predation in all seasons, as previously reported by Menezes-Netto et al. [30].

Egg masses were parasitized by *T*. *remus*, *Trichogramma* sp., and *Chelonus* sp. (Hymenoptera: Braconidae). The contribution of parasitoids to egg mortality was low (0–9%) in both release and non-release sites in all seasons, but there was a significant difference in *T*. *remus* parasitism (F_1,30_ = 15.04; *P* = 0.005) between sites with (1.92% ± 0.62) and without (0.82% ± 0.82) parasitoid release. The parasitism caused by *Trichogramma* sp. and *Chelonus* sp. also was not significantly different (F_1,30_ = 0.05; *P* = 0.83, and F_1,30_ = 0.00; *P* = 0.95, respectively) between treatments. The probability of dying from desiccation or unknown causes did not exceed 7%.

The highest rates of irreplaceable mortality were associated with egg inviability and dislodgment (wet seasons), and predation (dry seasons) (Table 3). For example, in the second season of 2010, if predators were eliminated from the mortality factors, mortality would be reduced by 11.3% in non-release fields and 13.6% in release fields, because that percentage of mortality would not have been replaced by other factors. Irreplaceable mortality from parasitism was very low (0–2.5%) compared with irreplaceable mortality from other factors.

**Table 3:** Total percentage mortality and irreplaceable mortality by year and seasons for FAW eggs in maize fields with and without *Telenomus remus* releases.

Year/Season	Percent total Mortality	Percent irreplaceable mortality					
		Predation	Parasitism	Dislodgement	Inviability	Desiccation	Unknown
**Without *T*. *remus***							
2009/2^nd^	71.8	7.8 (18.1)	0.4 (1.2)	1.6 (5.1)	43.2 (47.2)	0.7 (2.2)	0.1 (0.3)
2010/1^st^	68.3	2.5 (6.8)	0.4 (1.1)	17.0 (27.2)	24.1(32.7)	1.5 (4.4)	0.7 (2.0)
2010/2^nd^	77.1	11.3 (25.6)	0.5 (2.1)	1.8 (6.8)	35.9 (43.9)	0.2 (0.8)	0.5 (2.2)
2011/1^st^	70.6	28.2 (36.5)	1.0 (3.3)	3.9 (10.5)	13.8 (25.1)	0.1 (0.2)	0.1 (0.5)
2011/2^nd^	70.8	7.7 (17.5)	0.0 (0.1)	40.5 (44.9)	3.4 (9.5)	0.1 (0.2)	0.4 (1.4)
**With *T*. *remus* release**							
2009/2^nd^	77.5	4.3 (13.9)	2.5 (9.0)	1.4 (5.6)	46.4 (49.7)	0.7 (2.8)	0.1 (0.2)
2010/1^st^	65.8	2.8 (6.9)	0.5 (1.4)	17.7 (26.7)	19.7 (28.3)	2.8 (7.0)	1.1 (3.0)
2010/2^nd^	71.5	13.6 (25.3)	2.1 (6.4)	1.2 (4.0)	29.0 (37.2)	0.3 (1.2)	1.1 (3.5)
2011/1^st^	68.1	25.1 (33.6)	1.0 (2.9)	2.4 (6.6)	16.9 (27.1)	0.1 (0.3)	0.9 (2.7)
2011/2^nd^	72.2	4.4 (12.2)	1.5 (4.8)	44.8 (47.7)	2.7 (8.3)	0.3 (1.1)	0.4 (1.5)

Numbers in parentheses are the percentages of mortality in the presence of all other factors.

### Early larvae mortality

Six cohorts were used to develop partial multiple decrement life tables for early larvae (Table 4). The fate of several thousand individual larvae was investigated in each growing season (S2 Table). Greater than 95% of larvae died during the first seven days of the larval stage. The main factors contributing to this mortality included dislodgment by rainfall [31], drowning [31], predation [11], and perhaps neonate movement (larval ballooning) [32], here grouped as ‘other factors’. Mortality caused by these factors was irreplaceable in all seasons (Table 4).

**Table 4:** Multiple decrement life table and percentage irreplaceable mortality for early larval stage of FAW in maize fields.

Year/Season			Other Factors		Parasitism	
	aqx	Alx	aq1x	IM	aq2x	IM
2009/1st	0.973	1	0.973	97.3 (97.3)	0	0 (0)
2009/2^nd^	0.967	1	0.967	96.7 (96.7)	< 0.001	< 0.001
2010/1^st^	0.953	1	0.951	95.2 (95.5)	< 0.001	0.159
2010/2^nd^	0.995	1	0.995	99.5 (99.5)	< 0.001	0.012
2011/1^st^	0.981	1	0.981	98.1 (98.1)	< 0.001	0.012
2011/2^nd^	0.986	1	0.986	98.6 (98.6)	0	0 (0)

aq_x_—the probability of dying in stage x in the presence of all causes; al_x_—the fraction of the cohort living at the beginning of the stage; IM—percent of irreplaceable mortality. Numbers in parentheses are the percentages of mortality in the presence of other factors.

The most common predators found during plant inspection at the laboratory were *D*. *luteipes*, *H*. *axyridis*, *E*. *connexa*, *Orius insidiosus* (Say) (Hemiptera: Anthocoridae) and syrphid larvae (Diptera: Syrphidae). Parasitism was due to *Campoletis flavicincta* (Ashmead) (Hymenoptera: Ichneumonidae), *Ophion* sp. (Hymenoptera: Ichneumonidae), and tachinid flies (Diptera: Tachinidae).

When considering egg and larval mortality together, early larval mortality would have almost completely replaced egg mortality if the egg mortality factors were removed. This suggests that even if all eggs had survived, more than 95% of newly emerged larvae from those eggs would have died from predation, dislodgment, or drowning. The irreplaceable mortality of ‘other factors’ ranged from 22 to 32.1% (Table 5).

**Table 5:** Total percentage mortality and irreplaceable mortality by year and seasons when considering egg and larval mortality together in maize fields without and with *Telenomus remus* releases.

Year/	Percent total	Percent irreplaceable mortality						
Season	Mortality	Predation	Parasitism	Dislodgement	Inviability	Desiccation	Unknown	Other
**Without *T*. *remus***								
2009/2^nd^	99.074	0.255	0.012	0.052	1.421	0.021	0.002	27.241
2010/1^st^	98.517	0.116	0.019	0.793	1.112	0.072	0.03	30.193
2010/2^nd^	99.885	0.057	0.002	0.009	0.179	< 0.001	0.003	22.790
2011/1^st^	99.443	0.534	0.019	0.073	0.261	0.001	0.003	28.862
2011/2^nd^	99.603	0.104	< 0.001	0.549	0.046	< 0.001	0.006	28.885
**With *T*. *remus* release**								
2009/2^nd^	99.260	0.141	0.081	0.047	1.524	0.022	0.002	21.788
2010/1^st^	98.403	0.128	0.026	0.826	0.919	0.130	0.051	32.511
2010/2^nd^	99.858	0.068	0.010	0.006	0.145	0.002	0.005	28.312
2011/1^st^	99.397	0.475	0.019	0.046	0.320	0.002	0.017	31.239
2011/2^nd^	99.622	0.060	0.020	0.608	0.037	0.004	0.006	27.433

## Discussion

Demographic analytical methods were used to understand aspects of FAW mortality dynamics and irreplaceable mortalities, as well as to demonstrate the effect of each mortality factor on FAW populations. Abiotic factors had a greater effect on egg and early larval mortality, especially in rainy seasons, although predators also greatly reduced the number of small larvae. Furthermore, larval mortality was irreplaceable when compared to egg mortality. These results indicate that the larval stage might be a better target for control measures, because a greater population effect can be achieved with a small increment of mortality.

Our results reveal that rainfall seasonality influences the mortality dynamics of FAW eggs. During most rainy seasons, the contribution of predators to egg mortality was considerably lower than in dry seasons. Previous studies have shown that rain can limit the activity of natural enemies [33] and that *D*. *luteipes* in particular, the main predator of FAW egg masses, may have its foraging behavior negatively affected [34]. Additionally, egg dislodgement was more frequent in the rainy seasons than during in the dry seasons, indicating that rain and associated weather factors caused high rates of dislodgement.

Egg inviability was shown to be an important source of mortality in wet and dry seasons. According to Murúa et al. [35], the mean percentage of egg inviability is variable and ranges from 10 to 68% in natural FAW populations from Argentina. Similar data on Brazilian populations have not yet been assessed, but studies performed by Busato et al. [36] using lab-reared insects showed that 5 to 28% of FAW eggs were not viable. Indeed, in this study, egg masses that were kept in the laboratory to assess egg viability showed, on average, 15% of egg inviability. Egg masses that remained in the field for three days showed a higher degree of inviability, probably because they were exposed to hazards such as UV radiation that might have increased the level of unviable eggs [37]. It is also possible that some of the eggs considered unviable were actually parasitized, but parasitoids fail to develop and emerge. *Telenomus remus* emergence from FAW eggs is influenced by temperature, with emergence dropping from 96% to less than 6% when eggs are reared on 25°C or 34°C, respectively [38]. Because eggs remained in the field for several days, it is possible that high temperatures during the day might have reduced parasitoids survival.

In several occasions during the 2-h-long diurnal observations done in our experiments, more than one parasitoid was found parasitizing an egg mass concomitantly (S1 Fig). Competition between parasitoid larvae within the host egg can result in insect’s death [39]. In this study, mortality of parasitoid larvae would have been mistakenly accounted as egg inviability, because eggs were not dissected to assess parasitism. This could have caused an overestimation of egg inviability and an underestimation of the contribution of parasitoids to mortality of FAW eggs.

The low contribution of parasitoids to mortality of FAW eggs and larvae is most likely associated with a combination of factors. Intraguild predation, a trophic interaction between species using a shared prey resource [40], was observed among natural enemies of FAW. Previous studies on asymmetric intraguild predation have shown that some predators can consume parasitized or unparasitized eggs indiscriminately [41, 42], causing a negative effect on parasitism levels [43]. Moreover, the predation of parasitized eggs may be higher due to their longer time of exposure [44]. Host location also might have been negatively affected by the use of sentinel egg masses because they prevented oviposition-induced volatiles, often attractive to parasitoid species, to be released by plants [45], although other studies have shown a high percentage of *T*. *remus* parasitism on FAW sentinel egg masses [14, 19]. Also, it is possible that once the colony of *T*. *remus* has been reared in laboratory for many years, insects might have reduced their foraging ability and competitiveness.

For larvae mortality, Zalucki et al. [32] reported that more than 90% of Lepidoptera larvae can die during early larval stages because of natural enemies, dispersal or establishment, or weather or host-related factors. Fall armyworm larvae also can die due to cannibalism, although cannibalistic behavior is less frequent among early larvae [46]. Even with intense field observations, the fate of FAW larvae can be difficult to determine because of the habit of feeding deep in the whorl. Nevertheless, the diversity and abundance of predators found inside the whorl during plant inspection suggest that predators may have greatly contributed to FAW population suppression, as previously reported by Figueiredo et al. [11] and Wyckhuys and O’Neil [47]. It is important to note that due to neighboring plant removal, it was not possible to account for ballooning survivorship. Although neonate lepidopterans use silk strands to disperse, reduce competition, and increase survival, we are not aware of studies assessing ballooning survival of FAW in maize fields. A study on European Corn Borer, *Ostrinia nubilalis* (Hübner) (Lepidoptera: Crambidae) larvae has shown that only ca. 1% of the neonate larvae disperse to neighboring plants by ballooning in the first hours after emergence [48]. Therefore, we do not believe that the reductions in larval mortality due to ballooning would change the overall results obtained in these experiments. Early larva mortality was similarly high during wet and dry seasons and larval mortality likely would have an off-set effect on egg mortality. Therefore, a greater effect in reducing FAW generational survival may be achieved by adding mortality to early larval stages. Information provided by further studies performed on a landscape context, as shown by Wyckhuys and O’Neil [49], may help develop a sustainable method to conserve and increase FAWs predator populations in maize fields.

In conclusion, we showed that dynamics of FAW egg mortality factors are related mainly to inviability, rainfall, and predation, and that higher predation is associated with dry seasons. Cohort mortality levels and patterns did not change in areas with *T*. *remus* release and no parasitoid species caused appreciable mortality in eggs or early larval stages. Furthermore, the two native egg parasitoid species occurred in both sites, despite *T*. *remus* release. The irreplaceable mortality analytical technique within the multiple decrement life table revealed that larval mortality would almost completely replace egg mortality if the egg mortality factors were removed. Therefore, in maize fields, the early larval stage of FAW may be a more suitable target for control efforts. Such recommendation is based solely on FAW's mortality dynamics and it does not account for differences in the costs associated with incrementing larval versus egg mortality. Additional studies that aim to develop FAW biological control programs should consider the contribution of natural enemies, especially in dry seasons.

## Supporting Information

S1 Fig Sentinel fall armyworm egg masses in maize fields with augmentative release of *Telenomus remus*, Jaboticabal, São Paulo, Brazil, 2011. A) Sentinel egg mass C30 before a rainstorm; b) sentinel egg C30 mass after a rainstorm; c) sentinel egg mass B51 being parasitized by *T*. *remus* and *Trichogramma pretiosum*; d) close up of image C: solid arrows show *T*. *remus* and dashed arrow shows *T*. *pretiosum*; e) sentinel egg mass B51 after predation; f) close up of image E.
(TIF)

S1 Table Total number of *Spodoptera frugiperda* eggs evaluated for life table analysis. (XLSX)

S2 Table Total number of *Spodoptera frugiperda* larvae evaluated for life table analysis. (XLSX)
