# Supplementary figures and images for: Mortality Dynamics of Spodoptera frugiperda (Lepidoptera: Noctuidae) Immatures in Maize
Source: PLoS One. 2015 Jun 22;10(6):e0130437. doi: 10.1371/journal.pone.0130437 (PMC4476731; doi:10.1371/journal.pone.0130437)

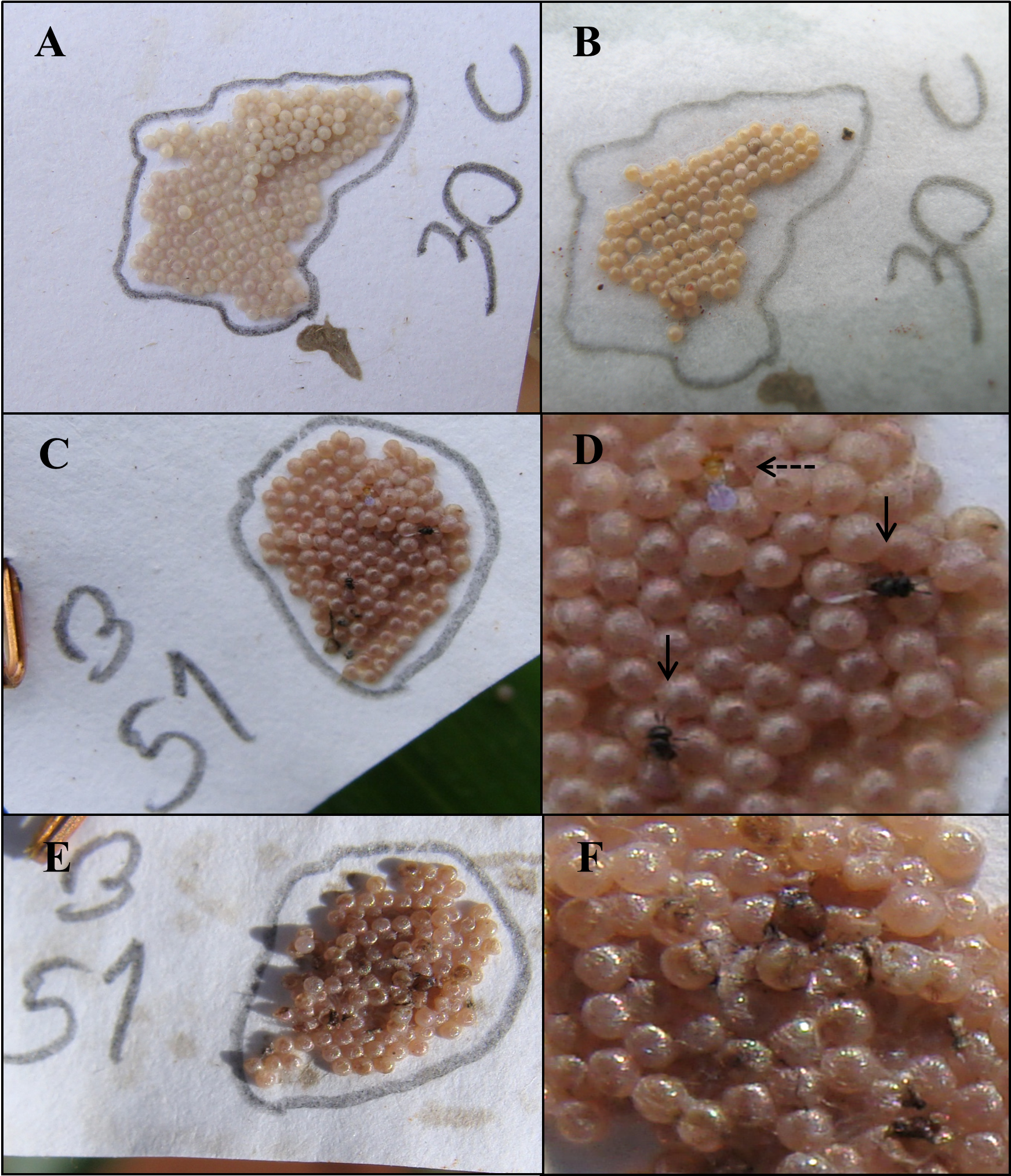

Supplement: S1 Fig — A) Sentinel egg mass C30 before a rainstorm; b) sentinel egg C30 mass after a rainstorm; c) sentinel egg mass B51 being parasitized by T. remus and Trichogramma pretiosum; d) close up of image C: solid arrows show T. remus and dashed arrow shows T. pretiosum; e) sentinel egg mass B51 after predation; f) close up of image E. (TIF) [file pone.0130437.s001.tif]
